# Water, sanitation, and hygiene and their association with child undernutrition: a hierarchical analysis of the 2020 Somaliland demographic health survey

**DOI:** 10.3389/fpubh.2025.1734101

**Published:** 2026-01-14

**Authors:** Hana Mahdi Dahir, Ayan Husein Korse, Farduus Ibraahim Mohamed

**Affiliations:** 1 School of Postgraduate Studies and Research (SPGSR), Amoud University, Borama, Somalia; 2 School of Postgraduate Studies and Research, University of Hargeisa, Hargeisa, Somalia

**Keywords:** child undernutrition, hierarchical analysis, multilevel logistic regression, stunting, wasting

## Abstract

**Background:**

Child undernutrition, manifested as stunting and wasting, remains a major public health concern globally, particularly in Somaliland. Inadequate Water, Sanitation, and Hygiene (WASH) are known contributors, their specific associations in Somaliland require investigation. This study examined the hierarchical relationships between WASH indicators and child undernutrition.

**Methods:**

We analyzed data from the 2020 Somaliland Demographic and Health Survey, including a weighted sample of 3,240 children aged 0–59 months. Stunting and wasting were defined using WHO 2006 Child Growth Standards. Household WASH indicators (latrine facility, drinking water source and water collection time) served as exposures. Multilevel logistic regression was used to assess associations, reporting Adjusted Odds Ratios (AORs) with 95% Confidence Intervals.

**Results:**

The prevalence of stunting was 23.4% and wasting 21.3%. Improved sanitation was associated with lower odds of stunting (AOR = 0.69; 95% CI: 0.56–0.86), while unimproved sanitation increased the likelihood of wasting (AOR = 1.30; 95% CI: 1.02–1.68). Children in households requiring less than 30 min to collect water had higher odds of stunting compared with those with water on premises (AOR = 1.39; 95% CI: 1.11–1.75). Female children were more likely to be wasted, and significant regional disparities in stunting were observed.

**Conclusion:**

Child undernutrition in Somaliland remains high and is strongly influenced by sanitation access, water-collection burden, and regional inequalities. Strengthening household sanitation and improving water accessibility are essential to reduce child undernutrition.

## Introduction

Child undernutrition, encompassing stunting, wasting, and underweight, remains one of the most critical public health challenges globally, accounting for nearly 45% of deaths among children under 5 years of age (1). In 2022, an estimated 148 million children under 5 were stunted and 45 million were wasted worldwide, reflecting a still very high global burden of undernutrition Although global efforts have reduced child undernutrition over recent decades, progress has been slow, and the burden remains disproportionately concentrated in low-income and fragile settings (2, 3). Child undernutrition, particularly stunting, remains a significant global health burden, affecting approximately 144 million children under 5 years of age worldwide (4). Stunting, a chronic condition marked by impaired growth and development, reflects poor health, nutritional deficiencies, and inadequate environmental conditions, with lasting consequences for cognition, education, and economic productivity (5).

Child wasting, a severe form of undernutrition characterized by low weight-for-height, continues to be a significant public health challenge globally, particularly in vulnerable regions such as Somaliland (6). Wasting contributes to nearly 45% of child mortality worldwide and affects about 45 million children under five, mainly due to poor dietary intake, illness, and limited food access. Beyond these immediate causes, inadequate WASH conditions—such as unsafe water, poor sanitation, and unhygienic environments—further increase vulnerability by driving recurrent enteric infections and diarrheal diseases, which are key pathways linking WASH deficiencies to undernutrition (7, 8).

In regions such as South Asia and Sub-Saharan Africa, where the burden of stunting is particularly high, understanding its multifactorial etiology, including environmental factors, is crucial for developing effective interventions (9). A growing body of research highlights the strong epidemiological association between access to adequate Water, Sanitation, and Hygiene and improved nutritional outcomes in children (4). Specifically, poor WASH conditions are estimated to contribute to 56% of malnutrition cases, influencing both the incidence of stunting and wasting in preschool-aged children (6, 10). This study conducted in Bangladesh reveals that poor WASH conditions are significantly associated with higher rates of malnutrition among children, as inadequate water and sanitation facilitate infections that impair nutrient absorption and growth, thereby contributing to the persistent burden of child malnutrition in the country (11). The study revealed that the prevalence of double burden of malnutrition at household level varies significantly among Bangladesh, Nepal, Pakistan, and Myanmar, with higher rates observed in urban areas and among households with middle to higher socioeconomic status (12). Similarly, a study in African countries of Gabon, Gambia, Liberia, Mauritania, and Nigeria found that key household factors such as household wealth, access to clean water and sanitation, maternal education, and recent child illnesses significantly influence the likelihood of child malnutrition (13).

This underscores the necessity of investigating the complex interplay between WASH indicators and various forms of child undernutrition, including stunting, underweight, and wasting, within specific geographic and socioeconomic contexts (14). Recent evidence from a multilevel analysis across 27 countries found that childhood malnutrition clustered geographically, with community-level WASH and socioeconomic conditions—such as poor sanitation, unsafe water, illiteracy, and unemployment—explaining nearly twice the variation in malnutrition compared to country-level factors (15).

In Somaliland, recent studies report a very high burden of child malnutrition, with up to 42% stunted, 30.4% underweight, and 49.3% wasted among children aged 6–59 months in Burao, and about 5% of primary school children in Hargeisa severely undernourished, highlighting a persistent public health crisis (16, 17). Recent local evidence from Somaliland found that the prevalence of stunting among children residing in internally displaced persons (IDP) camps in Hargeisa was 21.1%, with significant risk factors including poor vaccination status, lack of deworming, home delivery, inadequate maternal nutrition, and measles infection—all of which highlight the complex interplay between healthcare access, maternal practices, and child nutrition in vulnerable settings. Findings from community- and school-based studies in Somaliland consistently report high levels of undernutrition, indicating that child malnutrition remains a persistent public health challenge across different population groups (18). This study aims to elucidate the hierarchical associations between WASH indicators and child undernutrition in Somaliland, leveraging data from the 2020 Demographic and Health Survey to inform targeted public health strategies (9, 19).

This study examines the links between household Water, Sanitation, and Hygiene (WASH) conditions—specifically type of latrine, source of drinking water and time to collect water—and child undernutrition outcomes such as stunting, wasting, and underweight, using a hierarchical modeling approach that considers both household and community-level influences (20). Water, sanitation, and hygiene (WASH) factors were examined as key exposures due to their strong association with child nutritional outcomes in socio-economically vulnerable settings. In Somaliland, widespread rural and nomadic living, poverty, displacement, recurrent droughts, and limited infrastructure restrict access to improved water and sanitation services, increasing children’s exposure to infections and environmental contamination. These pathways impair nutrient absorption and contribute to undernutrition, including underweight and stunting. Drawing on the Somaliland Demographic Health Survey 2020, it addresses gaps in understanding context-specific drivers of malnutrition and provides evidence to inform interventions (6). Particular attention is given to how unsafe water sources, unimproved sanitation, and poor stool disposal practices increase exposure to diarrheal diseases, while inadequate flooring and long distances to collect drinking water further exacerbate risks for poor growth outcomes (9, 21).

## Methods

### Study setting

**Figure 1:**
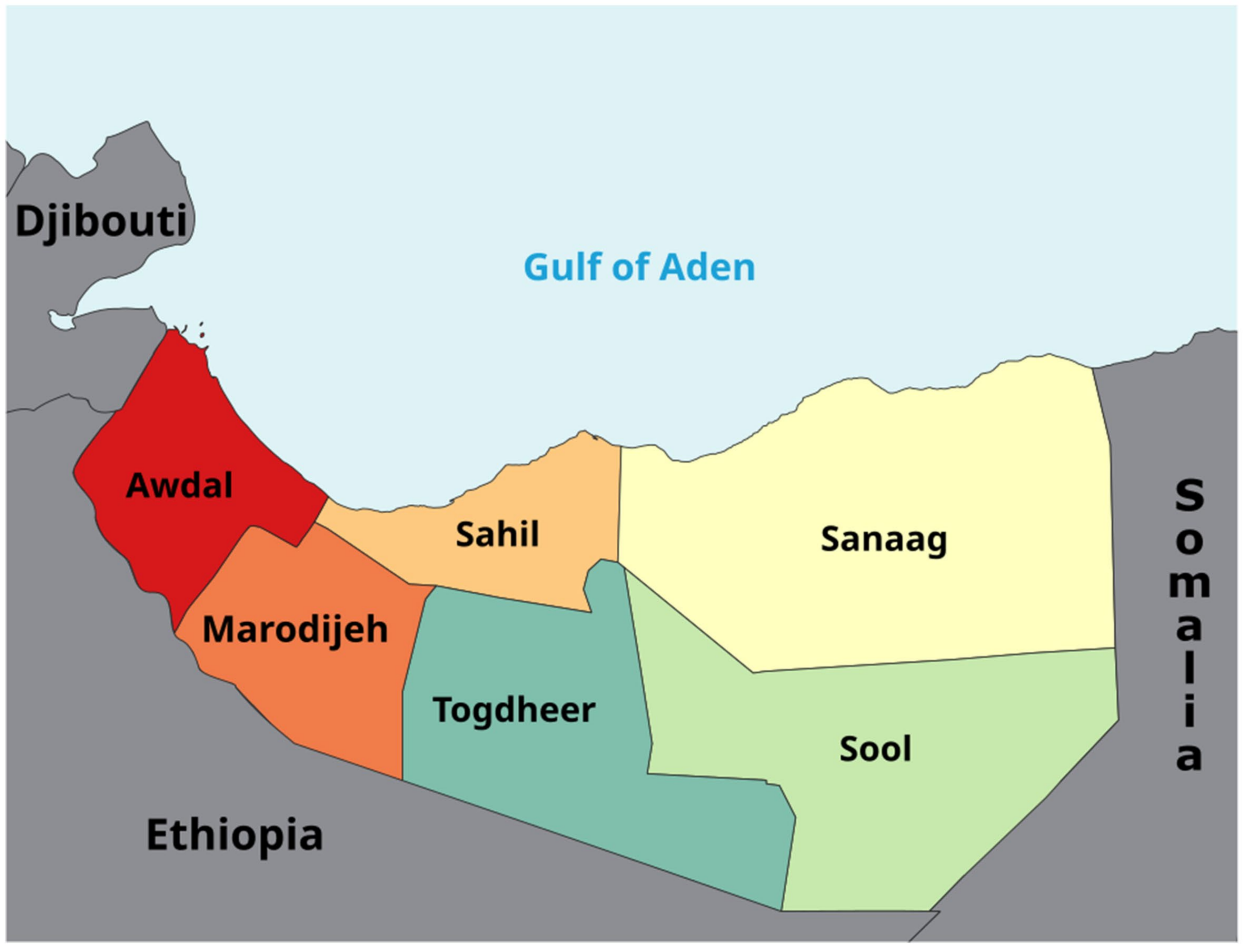
Map of Somaliland showing the study regions (Awdal, Sahil, Sanaag, Marodijeh, Togdheer, and Sool). Reproduced from “Map showing the administrative regions of Somaliland” by Jacob300, licensed under CC BY-SA 4.0.

The study was conducted in Somaliland, a self-declared state in the Horn of Africa with an estimated population of 5.7 million. Administratively, the country is divided into six regions: Awdal, Maroodijeex, Sahil, Togdheer, Sool, and Sanaag. These regions vary widely in socioeconomic conditions, geography, and access to health services. Undernutrition remains a major public health challenge across Somaliland, with persistently high rates of stunting, wasting, and underweight. Given the widespread reliance on unimproved water sources, poor sanitation facilities, and inadequate hygiene practices, Somaliland provides a critical context for examining the association between water, sanitation, and hygiene (WASH) indicators and child undernutrition (Figure 1).

### Data source

The primary data for this study were extracted from the 2020 Somaliland Demographic and Health Survey (SDHS). The SDHS is a nationally representative household survey implemented by the Central Statistics Department (CSD) of the Ministry of Planning and National Development, in collaboration with the Ministry of Health Development. To ensure high data quality and international comparability, the survey design and data collection procedures strictly followed the global USAID DHS Program protocols. Data were collected using Computer-Assisted Personal Interviewing (CAPI) on tablet computers, which included built-in logic and consistency checks. The survey collected detailed information on demographic characteristics, maternal and child health, nutrition, and household environmental factors. Standardized DHS questionnaires were administered, and anthropometric measurements of children under 5 years of age were taken using calibrated equipment and procedures consistent with World Health Organization (WHO) recommendations. The SDHS dataset provides an opportunity to assess child nutritional outcomes in relation to household and community-level WASH conditions.

### Sampling and data collection

The SDHS 2020 applied a two-stage stratified cluster sampling design. In the first stage, enumeration areas (EAs) were selected using probability proportional to size from the national census frame. In the second stage, households were systematically chosen from each EA. All eligible women aged 15–49 years residing in the selected households were interviewed, and anthropometric data were collected for their children under 5 years of age. For this study, children aged 0–59 months with complete anthropometric data and available household WASH information were included in the analysis, resulting in a weighted sample of 1,195.

### Study variables

#### Outcome variable

The outcome variables were three indicators of child undernutrition defined according to the WHO 2006 Child Growth Standards. Stunting was defined as a height-for-age z-score below minus two standard deviations, wasting as a weight-for-height z-score below minus two standard deviations. Each outcome was coded as a binary variable, with undernourished children coded as one and children with normal growth coded as zero. To ensure methodological transparency for, stunting (Height-for-Age) and wasting (Weight-for-Height) were calculated as Z-scores based on the WHO 2006 Child Growth Standards. Children with a Z-score below minus two standard deviations (−2 SD) from the median were classified as undernourished.

#### Independent variables

The independent variables for this study comprised household WASH characteristics and selected socio-demographic factors, chosen based on prior evidence of their association with child nutritional outcomes. Household WASH variables included the type of sanitation facility, categorized as improved, unimproved, or open defecation; source of drinking water, classified as improved or unimproved; and time required to collect drinking water, grouped as on premises, less than 30 min, or more than 30 min. Socio-demographic variables included sex of the child (male or female), age of the household head (categorized into standard DHS age groups), household wealth quintile (poor, middle, and rich), Place of Residence was categorized into Urban, Rural, and Nomadic settings. This three-way classification is critical in the Somaliland context which DHS was used, as nomadic populations face unique challenges regarding WASH infrastructure and food security that differ from settled rural or urban populations. This variable was used both as a descriptive measure and as a community-level covariate in the multilevel models to control for the confounding effect of residential setting on regional disparities.and region of residence. These variables were included to control for individual-, household-, and community-level factors that may confound the relationship between WASH conditions and child undernutrition.

### Statistical analysis

Data cleaning, recoding, and weighting were performed before analysis, and all statistical procedures were conducted using Stata version 17.0. Standard DHS weights were applied to the data to ensure the representativeness of the findings at the national and regional levels. Descriptive statistics were calculated to summarize sample characteristics and the prevalence of stunting and wasting. Logistic regression was used to assess associations between independent variables and child nutritional outcomes.

Given the hierarchical nature of the data, multilevel logistic regression models were employed to account for clustering of children within households and communities. Five models were fitted for each outcome variable. Model 0 was an empty model with no predictors, used to assess baseline variance and intra-class correlation coefficients (ICC). Model 1 combined individual-level characteristics with WASH indicators. Model 2 introduced community-level variables such as place and region of residence. Finally, Model 3 was the full model, which included WASH, individual, household, and community-level covariates. Results were reported as adjusted odds ratios (AOR) with 95% confidence intervals (CI). Model fit was evaluated using the Akaike Information Criterion (AIC), Bayesian Information Criterion (BIC), and log-likelihood statistics. Statistical significance was determined at *p* < 0.05.

## Results

Table 1 highlights substantial demographic, socioeconomic, and WASH-related disparities in child undernutrition in Somaliland. Regionally, the highest proportions of stunted children were observed in Sanaag (30.6%) and Sool (23.9%), while wasting was also most prevalent in Sanaag (27.5%) and Sool (22.6%), indicating marked geographic inequalities. Stunting was similarly distributed by child sex, with 48.0% among males and 52.0% among females, whereas wasting was more common among females (56.2%) than males (43.8%). With respect to household head age, children living in households headed by individuals aged 35–44 years accounted for the largest share of stunting (29.1%), while wasting was most frequently observed among households headed by individuals aged 25–34 years (22.8%) and ≥55 years (23.4%), suggesting differential vulnerability across household leadership age groups. Clear residential disparities were evident, with nomadic households contributing 41.1% of stunted children and 42.9% of wasted children, compared with urban households (33.3% stunting; 30.7% wasting). Sanitation conditions showed strong gradients, as households practicing open defecation accounted for nearly half of all stunted (49.6%) and wasted (50.9%) children, whereas improved sanitation facilities accounted for 28.4% of stunting and 26.7% of wasting. Similarly, 39.9% of stunted children lived in households using unimproved drinking water sources, and households requiring more than 30 min to fetch water accounted for 37.5% of stunting and 36.5% of wasting cases. Socioeconomic inequalities were pronounced, with children from poor households comprising 52.8% of stunted and 58.4% of wasted children, compared to those from rich households (32.8% stunting; 27.5% wasting). Overall, the descriptive statistics reveal substantial inequalities in child nutritional outcomes across regions, household head characteristics, residential setting, WASH conditions, and wealth status.

**Table 1:** Demographic distribution and prevalence of child stunting and wasting in Somaliland (2020 DHS).

Variable	Stunting prevalence (weighted %)		Wasting prevalence (weighted %)	
	No	Yes	No	Yes
	*N* (%)	*N* (%)	*N* (%)	*N* (%)
Regions				
Awdal	213.28 (8.60%)	48.587 (6.39%)	211.51 (8.30%)	50.35 (7.29%)
Maroodijeex	556.76 (22.45%)	146.90 (19.31%)	555.05 (21.77%)	148.61 (21.51%)
Sahil	123.76 (4.99%)	41.988 (5.52%)	138.02 (5.41%)	27.73 (4.01%)
Togdheer	492.89 (19.88%)	108.699 (14.29%)	483.56 (18.97%)	118.034 (17.08%)
Sool	448.64 (18.09%)	181.836 (23.90%)	474.29 (18.60%)	156.182 (22.60%)
Sanaag	644.46 (25.99%)	232.819 (30.60%)	687.27 (26.95%)	190.013 (27.50%)
Sex of child				
Male	1,199.38 (48.37%)	365.44 (48.03%)	1,261 (49.49%)	302.93 (43.84%)
Female	1,280.81 (51.63%)	395.39 (51.97%)	1,287 (50.51%)	388.1 (56.16%)
Mothers age				
<24	105.98 (4.27%)	34.51 (4.54%)	103.05 (4.04%)	37.45 (5.42%)
25–34	547.52 (22.08%)	141.32 (18.58%)	531.28 (20.84%)	157.56 (22.80%)
35–44	753.11 (30.37%)	221.48 (29.11%)	776.87 (30.47%)	197.72 (28.62%)
45–54	515.86 (20.80%)	156.59 (20.58%)	536.12 (21.03%)	136.33 (19.73%)
>55	557.33 (22.47%)	206.91 (27.20%)	602.38 (23.63%)	161.86 (23.43%)
Residence				
Urban	831.51 (33.53%)	253.011 (33.25%)	872.27 (34.21%)	212.25 (30.72%)
Rural	620.251 (25.01%)	265.301 (34.87%)	703.23 (27.58%)	182.31 (26.39%)
Nomadic	1,028 (41.06%)	242.525 (31.88%)	974.20 (38.21%)	296.36 (42.89%)
Toilet facility				
Improved	710.362 (28.65%)	286.80 (28.40%)	812.70 (31.87%)	184.46 (26.70%)
Un improved	540.07 (21.78%)	131.97 (17.35%)	517.63 (20.30%)	154.42 (22.35%)
Open defecation	1,229 (49.58%)	342.053 (44.96%)	1,219 (47.82%)	352.04 (50.95%)
Source of drinking water				
Un improved	1,419 (57.22%)	457.38 (60.12%)	1,475 (57.88%)	1,073 (42.12%)
Improved	1,060 (42.78%)	303.45 (39.88%)	400.569 (57.97%)	290.36 (42.03%)
Time to get water sources				
On premises	918.47 (37.04%)	253.68 (33.34%)	940.38 (36.88%)	231.77 (33.54%)
> 30 min	869.56 (35.07%)	285.25 (37.49%)	902.38 (35.39%)	252.42 (36.53%)
<30 min	691.78 (27.90%)	221.89 (29.17%)	706.94 (27.73%)	206.73 (29.92%)
Wealth quintile				
Poor	1,417 (57.16%)	401.712 (52.80%)	1,416 (55.54%)	403.24 (58.36%)
Middle	296.110 (11.94%)	109.781 (14.43%)	307.84 (12.07%)	98.046 (14.19%)
Rich	766.17 (30.90%)	249.346 (32.77%)	825.87 (32.39%)	189.64 (27.45%)

### Descriptive statistics

This table, Multilevel Logistics Regression Analysis of wasting presents the Adjusted Odds Ratios (AORs) and 95% Confidence Intervals (CIs) from a series of logistic regression models (Model 1 to Model 4) examining the association between various WASH factors and wasting, after controlling for confounders. In Model 1, individual and household-level WASH and socio-demographic variables were introduced. Unimproved latrine facilities were significantly associated with child wasting, with children in households using unimproved sanitation having 30% higher odds of wasting compared with those using improved sanitation (AOR = 1.30; 95% CI: 1.00–1.60; *p* = 0.036). Open defecation showed increased odds of wasting; however, the association was not statistically significant. Use of improved drinking water sources was associated with lower odds of wasting, but this relationship did not reach statistical significance. Similarly, time to fetch water, whether less than 30 min or more than 30 min compared with on-premises access, showed no significant association with wasting. Regarding socio-demographic factors, female children had significantly higher odds of wasting compared with males (AOR = 1.20; 95% CI: 1.00–1.40; *p* = 0.017). Children living in nomadic households also exhibited higher odds of wasting (AOR = 1.50; 95% CI: 0.80–2.70; *p* = 0.023). Household head age and wealth quintile were not significantly associated with wasting in this model.

Model 2 examined community-level characteristics while excluding individual-level variables. Regional variations in wasting were observed, but none of the regions showed statistically significant differences compared with the reference region. Place of residence (rural or nomadic), community poverty level, community water coverage, and community sanitation coverage were also not significantly associated with child wasting. Although some point estimates suggested higher risk in nomadic and high-poverty communities, the wide confidence intervals indicate substantial uncertainty. The full model (Model 3) simultaneously adjusted for both individual- and community-level variables. The association between unimproved sanitation and wasting remained statistically significant, confirming its independent effect (AOR = 1.30; 95% CI: 1.02–1.68; *p* = 0.031). In contrast, open defecation was not significantly associated with wasting after adjustment, suggesting that its effect may be mediated through other household or community conditions. The association between female sex and wasting remained significant in the full model (AOR = 1.19; 95% CI: 1.00–1.30; *p* = 0.022). The previously observed elevated odds among children from nomadic households were attenuated and no longer statistically significant after controlling for community-level factors. Drinking water source, time to water source, household wealth, household head age, region, and community poverty all remained non-significant predictors of wasting in the fully adjusted model.

Across all models, unimproved sanitation emerged as the most consistent and robust WASH-related predictor of child wasting, maintaining statistical significance even after accounting for community-level influences. Female children were also at significantly higher risk of wasting, highlighting important sex-based disparities in acute undernutrition. While other WASH components such as water source and access time showed protective trends, their associations were not statistically significant after multilevel adjustment. Community-level factors explained part of the between-community variation but did not independently predict wasting. These findings underscore the critical role of household sanitation improvements in reducing child wasting in Somaliland, while also emphasizing the need for targeted interventions addressing gender-related vulnerabilities (Table 2).

**Table 2:** Multilevel logistic regression analysis of WASH and socio-demographic factors linked to child wasting.

Variables	Model 0	Model 1	Model 2	Model 3
		AOR (95%CI) *p*-value	AOR (95%CI) *p*-value	AOR (95%CI) *p*-value
WASH Factors				
Latrine facility				
Improved		1		1
Un improved		1.3 (1.0–1.6) 0.036		1.3 (1.02–1.68)0.031
Open defecation		1.1 (0.85–1.6)		1.0 (73.-1.50)
Sources of drinking water				
Unimproved		1		1
Improved		0.89 (0.75–1.0)		0.92 (0.78–1.09)
Time to get water sources				
On premise		1		
> 30 min		0.94 (0.75–1.1)		0.92 (0.739–1.15)
<30 min		1.0 (0.84–1.3)		1.05 (0.834–1.33)
Place of resident				
Urban			1	1
Rural			1.13 (0.80–1.5)	1.1 (0.79–1.59)
Nomadic			1.50 (0.80–2.7) 0.023	1.5 (0.77–2.96)
Child sex				
Male		1		1
Female		1.20 (1.0–1.4) 0.017		1.19 (1.0–1.3) 0.022
Household head Age				
<24		1		1
25–34		0.89 (0.59–1.3)		0.89 (0.59–1.3)
35–44		0.86 (0.58–1.2)		0.86 (0.58–1.2)
45–54		0.82 (0.55–1.1)		0.82 (0.55–1.2)
≥55		0.90 (0.61–1.3)		0.89 (0.60–1.3)
Wealth quintile				
Poor		1		1
Middle		1.0 (0.75–1.4)		1.05 (0.75–1.4)
Rich		0.83 (0.60–1.1)		0.88 (0.62–1.2)
Regions				
Awdal			1	1
Marodijeh			0.93 (0.61–1.4)	0.94 (0.60–1.4)
Sahil			0.69 (0.45–1.0)	0.70 (0.46–1.1)
Togdheer			0.91 (0.62–1.3)	0.92 (0.61–1.3)
Sool			1.2 (0.88–1.7)	1.2 (0.88–1.7)
Sanaag			0.98 (0.69–1.3)	1.01 (0.70–1.4)
Community Poverty				
Low			1	
High			0.91 (0.48–1.71)	0.87 (0.45–1.68)

Based on Table 3, Model 0 (empty model) shows significant community-level variation in wasting, with an ICC of 7.6%, indicating that a meaningful proportion of variability is attributable to differences between clusters. Model 1, which includes individual-level factors, slightly reduces the ICC and variance, suggesting that child- and household-level characteristics explain part of this clustering, although overall model fit does not substantially improve as reflected by a higher AIC and BIC. Model 2, which incorporates community-level factors, demonstrates the best model fit, as it has the lowest AIC (4264.643) and BIC (4327.952), along with a reduced ICC (6.7%) and lower random-effect variance. This indicates that community characteristics contribute importantly to explaining variation in child wasting and do so more efficiently than individual factors alone. Model 3 (full model) achieves the highest log likelihood, but its higher AIC and BIC indicate overfitting without proportional improvement in explanatory power. Overall, Model 2 is the best and most parsimonious model for explaining child wasting, balancing goodness of fit with model simplicity while adequately accounting for community-level variation.

**Table 3:** Model comparison and random effect analysis result (wasting).

Parameters	Model 0	Model 1	Model2	Model 3
AIC	4265.509	4269.647	4264.643	4272.547
BIC	4278.17	4358.279	4327.952	4411.826
Log likelihood	−2130.7543	−2120.8236	−2122.3216	−2114.2736
ICC	0.0757321	0.0732027	0.0673602	0.0696615
Variance	0.2695633	0.2598488	0.2376117	0.2463375

Table 4 presents the results of a multilevel logistic regression analysis evaluating the associations between WASH and socio-demographic factors and child stunting. In the fully adjusted model (Model 3), several WASH factors emerged as significant predictors of nutritional status. Specifically, children living in households with improved latrine facilities had significantly lower odds of stunting compared to those using unimproved facilities (AOR = 0.693; 95% CI: 0.557–0.862; *p* = 0.001), highlighting a strong protective effect of sanitation. Access to water was also a significant factor, as children in households requiring less than 30 min to collect water had 1.39 times higher odds of stunting compared to those with water on the premises (AOR = 1.39; 95% CI: 1.11–1.75; *p* = 0.004). However, the type of drinking water source did not show a statistically significant association with stunting after full adjustment.

**Table 4:** Multilevel logistic regression analysis of WASH and socio-demographic factors linked to child stunting.

Variables	Model 0	Model 1	Model 2	Model 3
		AOR (95%CI) *p*-value	AOR (95%CI) *p*-value	AOR (95%CI) *p*-value
WASH Factors				
Latrine facility				
Un Improved		1		1
Improved		0.893 (0.757–1.05) 0.000		0.693 (0.557–0.862) 0.001
Sources of drinking water				
Un Improved		1		1
Improved		0.893 (0.757–1.05)		935 (0.791–1.10)
Time to get water sources				
On premise		1		1
> 30 min		1.23 (0.997–1.53)		1.23 (0.995–1.535)
<30 min		1.36 (1.08–1.70) 0.007		1.39 (1.11–1.75) 0.004
Place of resident				
Urban			1	1
Rural			0.954 (−0.662 1.38)	918 (0.632–1.33)
Nomadic			0.748 (0.307–1.822)	0.716 (0.288–1.78) 0.001
Child sex				
Male		1	1	1
Female		1.029 (0.886–1.19)		1.01 (0.874–1.18)
Age of household head				
≤24		1	1	1
25–34		0.811 (0.543–1.2)		0.796 (0.533–1.19)
35–44		0.933 (0.632–1.37)		0.906 (0.616–1.348)
45–54		0.876 (0.588–1.30)		0.851 (0.575–1.27)
≥55		0.942 (0.635–1.39)		0.851 (0.620–1.36)
Wealth quintile				0.919 (0.618–1.36)
Poor		1	1	1
Middle		0.955 (0.721–1.26)		0.830 (0.619–1.11)
Rich		1.022 (0.775–1.34)		0.876 (648–1.184)
Regions				
Awdal			1	1
Marodijeh			1.01 (0.630–1.63)	1.014 (0.626–1.64)
Sahil			0.943 (0.584–1.52)	0.898 (0.552–1.46)
Togdheer			1.02 (0.6526–1.61)	0.921 (0.579–1.46)
Sool			1.83 (1.24–2.69) 0.002	1.69 (1.14–2.52) 0.007
Sanaag			1.80 (1.22–2.66) 0.003	1.60 (1.14–2.52) 0.013
Community Poverty				
Low			1	1
High			0.78 (0.34–1.96)	0.77 (0.303–1.95)

Regarding socio-demographic and geographic variables, the place of residence showed that children from nomadic backgrounds had significantly lower odds of stunting compared to urban residents (AOR = 0.716; 95% CI: 0.288–1.78; *p* = 0.001). Regional disparities were also prominent; children residing in the Sool (AOR = 1.69; 95% CI: 1.14–2.52; *p* = 0.007) and Sanaag (AOR = 1.60; 95% CI: 1.14–2.52; *p* = 0.013) regions experienced significantly higher odds of stunting compared to those in the Awdal region. Other factors, including child sex, age of the household head, household wealth quintile, and community-level poverty, were not found to be statistically significant predictors of stunting in the final model (*p* > 0.05). These findings suggest that improving sanitation infrastructure, reducing water-collection burdens, and targeting regional inequalities are essential strategies for reducing chronic undernutrition in children.

Table 5 presents a comparison of four multilevel logistic regression models for child stunting. Model 0 serves as a baseline, and Models 1–3 incorporate individual-, household-, and community-level predictors. Model fit improves across models, evidenced by decreasing AIC values from 4455.161 in Model 0 to 4432.278 in Model 3, indicating enhanced explanatory power. The log likelihood becomes less negative, reaching its peak in Model 3 (−2195.14). Although BIC values are lower for Model 0 due to penalization for added parameters, more complex models generally show higher BIC values. The intra-class correlation coefficient (ICC) remains around 20%, suggesting substantial variation in stunting between clusters. However, the ICC slightly decreases from 0.22 in Model 0 to 0.20 in Model 3, indicating that included factors explain some between-cluster variability. Cluster-level variance also decreases from 0.92 in Model 0 to 0.82 in Model 3, confirming that the predictors reduce unexplained community-level differences while still leaving some residual variation. In conclusion, Model 3 is the best-fitting model with the lowest AIC and highest log likelihood, validating the use of multilevel modeling for analyzing child stunting.

**Table 5:** Model comparison and random effect analysis result (stunting).

Parameters	Model 0	Model 1	Model 2	Model 3
AIC	4455.161	4446.288	4434.334	4432.278
BIC	4467.823	4528.589	4497.643	4565.226
Log likelihood	−2225.5804	−2210.1439	−2207.1672	−2195.1388
ICC	0.2193544	0.2151144	0.1968757	0.1999292
Variance	0.9244234	0.9016575	0.8064694	0.822103

## Discussion

This study utilized a multilevel analysis of the 2020 Somaliland Demographic and Health Survey to explore the determinants of child undernutrition. Our findings reveal a stunting prevalence of 23.4% and a wasting prevalence of 21.3% among children under five. This indicates a high burden of undernutrition; the wasting rate, in particular, exceeds the WHO “critical” threshold of 21.3% and is significantly higher than the global average of 6.7% (22). These rates are comparable to regional neighbors like Ethiopia (stunting 23.1%) and Kenya, where stunting has been shown to rise significantly as children reach 15 months of age (23, 24).

The regression analysis demonstrates that sanitation is a primary driver of both chronic and acute undernutrition in Somaliland. Unlike previous hypothetical drafts suggesting an “unexpected” protective effect of open defecation, the actual data confirms that improved sanitation is protective. Children in households with improved latrines had significantly lower odds of stunting (AOR = 0.693; *p* = 0.001), while those using unimproved facilities faced a 30% increase in the odds of wasting (AOR = 1.30; *p* = 0.031). These results are consistent with findings in Ethiopia, where unimproved sanitation significantly increased stunting risks (AOR = 1.29) (25), and in China, where WASH conditions were a major mediator of nutritional disparities (25). Our findings support the global consensus that improved household latrines reduce the risk of environmental enteric dysfunction, thereby protecting children from both stunting and wasting (19, 26).

Regarding water access, the study found that the time burden of water collection is more significant than the water source itself for chronic undernutrition. Children in households requiring less than 30 min to collect water had 1.39 times higher odds of stunting compared to those with water on the premises (AOR = 1.39; *p* = 0.004). This aligns with research in Ibadan, Nigeria, where longer water sourcing times were significantly associated with poorer nutritional outcomes (27). Interestingly, while water collection time was a risk factor for stunting, it did not reach statistical significance for wasting in the final adjusted model. This suggests that the time-poverty associated with water collection may have a cumulative effect on child care and hygiene practices over time, leading to chronic growth failure (stunting) rather than acute weight loss (wasting).

Socio-demographic factors revealed unique regional and gender-based vulnerabilities. Female children in Somaliland were found to have significantly higher odds of wasting than males (AOR = 1.19; *p* = 0.022), a finding that suggests gender-specific differences in acute nutritional vulnerability or care-seeking behaviors. Furthermore, significant geographic inequalities were observed; children in the Sool (AOR = 1.69; *p* = 0.007) and Sanaag (AOR = 1.60; *p* = 0.013) regions were at the highest risk for stunting. This regional variation mirrors findings in Pakistan and Nigeria, where geographic location and local infrastructure often outweigh individual household wealth in predicting child health outcomes (27, 28).

A notable finding in this study was that nomadic residence appeared protective against stunting compared to urban residence (AOR = 0.716; *p* = 0.001). This contrasts with the typical urban–rural disparity seen in countries like China, where urban areas generally fare better (29). This unique result in Somaliland may be due to the nomadic population’s access to animal-source foods, such as milk and meat, which are critical for preventing chronic growth failure, despite the lack of permanent WASH infrastructure.

In conclusion, while the burden of undernutrition in Somaliland remains high, the drivers are clearly linked to sanitation access, water-collection burdens, and regional disparities. Interventions should focus on expanding improved latrine coverage and bringing water sources closer to the home, with a specific focus on the highly vulnerable Sool and Sanaag regions.

### Recommendations

Based on the findings, the Somaliland Ministry of Health Development, in collaboration with UNICEF and other stakeholders, should prioritize interventions that address critical WASH deficiencies directly linked to child undernutrition. Given the strong association between longer water collection times and increased child wasting, immediate efforts should focus on improving access to safe drinking water sources within or very close to households, potentially through community water points, boreholes, or piped water systems. Additionally, the consistently high odds of wasting linked to unsafe child stool disposal demand targeted public health campaigns on proper child faeces management, including safe disposal practices and the importance of latrine use for young children. While the protective effect of open defecation against stunting found in this study is unexpected and warrants further qualitative and contextual investigation to understand underlying factors, the overall emphasis should remain on promoting improved sanitation facilities. This could involve community-led total sanitation (CLTS) approaches adapted to local cultural norms, alongside hygiene education programs emphasizing handwashing with soap at critical times, especially before food preparation and after defecation, to break the faecal-oral transmission routes that contribute to malnutrition. Finally, given the observed regional disparities and socioeconomic determinants, interventions should be geographically targeted to vulnerable regions like Sanaag and prioritize poorer households, integrating WASH improvements with broader maternal and child health and nutrition programs.

## Conclusion

This study demonstrates that child undernutrition in Somaliland remains unacceptably high and is strongly influenced by household water and sanitation conditions, alongside socio-demographic and regional factors. Improved sanitation was consistently associated with reduced odds of stunting, while unimproved sanitation increased the risk of wasting. Limited access to drinking water, particularly the time required to collect water, was an important determinant of chronic undernutrition. In addition, notable regional disparities highlight the influence of community-level conditions on child nutritional outcomes.

These findings underscore the importance of integrating WASH interventions with child nutrition programs to address both chronic and acute forms of undernutrition. Priority should be given to improving sanitation infrastructure, reducing the burden of water collection, and implementing region-specific strategies targeting the most affected areas. Strengthening multisectoral approaches that combine WASH, nutrition, and social development initiatives is essential to improve child health and survival in Somaliland.

## Data Availability

The data analyzed in this study is subject to the following licenses/restrictions: the data used in this study were obtained from the Central Statistics Department, Ministry of Planning, Somaliland. The dataset is not publicly available due to institutional restrictions but can be accessed upon reasonable request by contacting the Central Statistics Department, Ministry of Planning, Somaliland. Approval and consent to participate. Requests to access these datasets should be directed to admin@somalilandcsd.org.
